# The Torpid State: Recent Advances in Metabolic Adaptations and Protective Mechanisms^†^

**DOI:** 10.3389/fphys.2020.623665

**Published:** 2021-01-20

**Authors:** Sylvain Giroud, Caroline Habold, Roberto F. Nespolo, Carlos Mejías, Jérémy Terrien, Samantha M. Logan, Robert H. Henning, Kenneth B. Storey

**Affiliations:** ^1^Research Institute of Wildlife Ecology, Department of Interdisciplinary Life Sciences, University of Veterinary Medicine, Vienna, Austria; ^2^University of Strasbourg, CNRS, IPHC, UMR 7178, Strasbourg, France; ^3^Instituto de Ciencias Ambientales y Evolutivas, Universidad Austral de Chile, ANID – Millennium Science Initiative Program-iBio, Valdivia, Chile; ^4^Center of Applied Ecology and Sustainability, Departamento de Ecología, Facultad de Ciencias Biológicas, Pontificia Universidad Católica de Chile, Santiago, Chile; ^5^Unité Mécanismes Adaptatifs et Evolution (MECADEV), UMR 7179, CNRS, Muséum National d’Histoire Naturelle, Brunoy, France; ^6^Department of Biology, Carleton University, Ottawa, ON, Canada; ^7^Department of Clinical Pharmacy and Pharmacology, University Medical Center Groningen, Groningen, Netherlands

**Keywords:** body temperature, metabolic depression, hibernation, hormones, lipids, non-Holarctic heterotherms, antioxidant, H_2_S

## Abstract

Torpor and hibernation are powerful strategies enabling animals to survive periods of low resource availability. The state of torpor results from an active and drastic reduction of an individual’s metabolic rate (MR) associated with a relatively pronounced decrease in body temperature. To date, several forms of torpor have been described in all three mammalian subclasses, i.e., monotremes, marsupials, and placentals, as well as in a few avian orders. This review highlights some of the characteristics, from the whole organism down to cellular and molecular aspects, associated with the torpor phenotype. The first part of this review focuses on the specific metabolic adaptations of torpor, as it is used by many species from temperate zones. This notably includes the endocrine changes involved in fat- and food-storing hibernating species, explaining biomedical implications of MR depression. We further compare adaptive mechanisms occurring in opportunistic vs. seasonal heterotherms, such as tropical and sub-tropical species. Such comparisons bring new insights into the metabolic origins of hibernation among tropical species, including resistance mechanisms to oxidative stress. The second section of this review emphasizes the mechanisms enabling heterotherms to protect their key organs against potential threats, such as reactive oxygen species, associated with the torpid state. We notably address the mechanisms of cellular rehabilitation and protection during torpor and hibernation, with an emphasis on the brain, a central organ requiring protection during torpor and recovery. Also, a special focus is given to the role of an ubiquitous and readily-diffusing molecule, hydrogen sulfide (H_2_S), in protecting against ischemia-reperfusion damage in various organs over the torpor-arousal cycle and during the torpid state. We conclude that (i) the flexibility of torpor use as an adaptive strategy enables different heterothermic species to substantially suppress their energy needs during periods of severely reduced food availability, (ii) the torpor phenotype implies marked metabolic adaptations from the whole organism down to cellular and molecular levels, and (iii) the torpid state is associated with highly efficient rehabilitation and protective mechanisms ensuring the continuity of proper bodily functions. Comparison of mechanisms in monotremes and marsupials is warranted for understanding the origin and evolution of mammalian torpor.

## Introduction

Torpor and hibernation represent powerful strategies that enable animals to survive periods of low resource availability in their environment. The state of torpor results from an active and drastic reduction of metabolic rate (MR) associated with an accompanying decrease in body temperature (T_b_) after passive cooling (Heldmaier et al., 2004; Jastroch et al., 2016). According to several authors (Ruf and Geiser, 2015; Geiser, 2020), two different forms of torpor, also called heterothermy, can be distinguished based on the duration of the hypometabolic state. In daily torpor, animals show average minimum torpid MR of ∼19% of basal rates and lower their T_b_ to usually between 12 and 25°C during torpor; torpor lasts less than 24 h and is accompanied by continued foraging (Ruf and Geiser, 2015). On the other hand, during hibernation, individuals achieve a minimum torpid MR of 4% of basal rates, along with a variable reduction of their T_b_ ranging on average for most species between 0 and 10°C (Ruf and Geiser, 2015). Hence, hibernation corresponds to multiple and successive torpor bouts lasting for days to few weeks, during which animals rely entirely on fuel stores, such as body fat and/or food caches (Humphries et al., 2003a,b; Dark, 2005). Torpor is employed by all three mammalian subclasses, i.e., monotremes, marsupial, and placentals, as well as several avian orders (Ruf and Geiser, 2015) whereas hibernation is documented in mammals from all three subclasses but is known for only one bird species. Daily torpor is diverse in both mammals and birds, and is typically not as seasonal as hibernation (Geiser, 2020). The use of torpor is often associated with species inhabiting cold and seasonal habitats, such as temperate and arctic zones, but torpor is also used by many non-Holarctic species, i.e., the tropics and southern hemisphere (Nowack et al., 2020).

Most seasonal heterothermic eutherian species enter an obligatory physiological and behavioral preparation period when days become shorter, i.e., announcing the winter season. Such remodeling is controlled by the hypothalamus, operates at multiple levels (reviewed in Auld et al., 2010), and comprises a variety of physiological, biochemical, and molecular changes such as regulations of metabolic processes, erythropoiesis, protein transcription, membrane composition, and thermogenesis (Klug and Brigham, 2015; Ruf and Geiser, 2015; Jastroch et al., 2016). This leads to drastic changes in most of biological functions, including metabolic (Jastroch et al., 2016) and reproductive (Clarke and Caraty, 2013) signaling pathways. Actually, the physiological preparations for harsh winter conditions is probably explained by an adjustment of the thermoneutral zone combined with a decrease in basal MR (Kobbe et al., 2014), which contributes to the accumulation of energy body (fat) stores. Additionally, heterothermic species adjust the onset of torpor or hibernation periods to environmental factors, including food availability and/or quality (notably in terms of lipid composition), and T_a_ (Boyles et al., 2007; Geiser, 2013; Giroud et al., 2018), therefore introducing some flexibility to these syndromes. Further, diverse phenology of hibernation was described among different age classes, i.e., juveniles, yearlings, and adults (Michener, 1978; French, 1990; Kart Gür and Gür, 2015; Siutz et al., 2016), but also in aged individuals compared to younger ones (e.g., Bieber et al., 2018). These differences were mostly linked to ecological cues, including developmental constraints, rather than physiological or molecular mechanistic aspects. For instance, it is known that juveniles, especially those born late in the reproductive season, delay hibernation onset compared to yearlings or adult individuals (Boag and Murie, 1981; Stumpfel et al., 2017), due to their need to further grow and develop and extra time needed to accumulate sufficient fat reserves prior to hibernation (Mahlert et al., 2018); a process that strongly determines the overwintering survival of the individuals. The demonstration that the use of torpor and the physiological remodeling required for the setup of the torpor phenotype is a deeply conserved feature in species living in seasonal environments comes from the variety of studies both from wild and captive conditions. Indeed, organisms maintained under captive environment, with stable and favorable conditions, show persistent circannual rhythms even after more than fifty generations (Perret and Aujard, 2001; Terrien et al., 2017), indicating the deep genetic imprinting of the control of biological clocks on metabolism and reproduction (Dardente et al., 2019).

During the hysteresis of the torpor-arousal cycle over hibernation, the whole organism including organs, tissues, cells, and molecules experience major metabolic transitions triggered by extreme changes in MR and T_b_. This is notably the case during the transition phase of arousal from torpor when individual’s MR shows a drastic several-fold increase allowing T_b_ to return to normothermia. Periodic arousals represent the highest proportion of energy expended during the hibernation process, e.g., 70–80% in temperate species (Wang, 1978), and arousals are accompanied with increases in oxidative stress levels, as supported by substantial shortening of telomere lengths in individuals over winter hibernation (Turbill et al., 2013; Giroud et al., 2014; Hoelzl et al., 2016; Nowack et al., 2019). Prior to hibernation, antioxidant defenses, and other specific regulatory processes are also upregulated to ensure the integrity of body organs, tissues, and cells (for review, see Carey et al., 2003). During the torpid state, specific protective mechanisms enter into play to ensure the continuity of key physiological functions, including heart, lung, muscle, and brain activity, to sustain life at low pace (for reviews, see Johansson, 1996; Ruf and Arnold, 2008; Talaei et al., 2012; Jastroch et al., 2016; Chanon et al., 2018; Nespolo et al., 2018).

Despite the threatening side associated with the torpid state, some progress has been made to further understand the mechanisms underlying the onset, maintenance and arousal from torpor, notably by pharmacological induction of hypometabolism in non-heterothermic species (Bouma et al., 2012). Several molecules and compounds for the induction and the maintenance of a torpor-like state in non-heterotherms have so far received special attention, including 5′-AMP and hydrogen sulfide (H_2_S). In particular, the latter seems to be involved in the metabolic maintenance and protective mechanisms of the torpid state in hibernators (see section below for further details). Recently, it has been established that fasting and cold are the most important proximate determinants of torpor in mice, acting by a hypothalamic neuronal circuit as a feedback between fasting, cold, and brown adipose tissue (BAT) activity. Using “designer receptors exclusively activated by designer drugs” (DREADDs) systems, specific hypothalamic neurons were identified and were shown to be involved in the occurrence of either a short-term (daily) torpor (Hrvatin et al., 2020) or a long-term, multi-days hypometabolic state in mice similar to hibernation (Takahashi et al., 2020). Interestingly, a light-sensitive receptor protein expressed in neurons of the preoptic area inhibits BAT metabolism, thus demonstrating a strong link between light and metabolism in animals (Zhang et al., 2020). These results, however, open the question as to how this could work in heterothermic animals with no functional descriptions of BAT, i.e., marsupials and monotremes (Gaudry and Campbell, 2017). One mechanism and its evolutionary implications for heat generation as an alternative to uncoupling processes in BAT could be linked to non-shivering thermogenesis (NST) in muscle (reviewed in Nowack et al., 2017). Recently, this has been demonstrated to occur in wild boar piglets, a species lacking BAT, but which demonstrates marked thermoregulatory adaptive responses (Nowack et al., 2019).

The objective of the present review is then to highlight some of the characteristics associated with the torpor phenotype. In the first section, we focus on specific adaptations for torpor occurring in heterotherms that show contrasting heterothermic patterns. This includes (i) metabolic and endocrine changes involved in fat-storing vs. food-storing hibernating species, and (ii) adaptive mechanisms occurring in opportunistic vs. seasonal heterotherms, such as tropical and sub-tropical species. The latter notably brings, based on recent data on molecular aspects, new insights on the metabolic origins of hibernation among tropical species, and on the view of daily torpor and hibernation as a continuum of hypometabolic responses. In the second section of this review we emphasize the molecular mechanisms enabling heterotherms to protect key organs against potential threats, such as reactive oxygen species, associated with the torpid state. We notably address mechanisms of cellular rehabilitation and protection during torpor and hibernation, with (i) an emphasis on the brain, a central organ to be protected during torpor and recovery after, and (ii) the role of a ubiquitous and readily-diffusing molecule, H_2_S, in protecting the integrity of organs against damage occurring over the torpor-arousal cycle and during the torpid state.

## Metabolic Adaptations During Torpor and Hibernation

### Fat-Storing vs. Food-Storing Hibernators: Hibernating Patterns, Lipid, and Energy Metabolism

Hibernators are generally classified as either (i) fat-storing species, i.e., animals that do not feed during hibernation and rely entirely on body fat reserves accumulated prior to hibernation to cover winter energy requirements, or (ii) food-storing species that feed during periodic arousals between deep torpor bouts and therefore hoard large amounts of food prior to winter (mainly seeds) in their burrow (French, 1988). Due to these different strategies of energy acquisition, fat- and food-storing species show contrasting physiological and behavioral adaptations that we will review in this section.

Fat-storing species typically undergo an intense period of hyperphagia to build up large amounts of internal fat reserves during several weeks to months before hibernation. As example, golden-mantled ground squirrels (*Callospermophilus lateralis*) show respectively a two and threefold increase in body and fat mass, in only 5–7 weeks prior to hibernation (Kenagy and Barnes, 1988). Yellow-bellied marmots (*Marmota flaviventris*) also increase their body mass up to 150% through accumulating 3–4.5 kg body fat during their active period in summer (Florant et al., 2004). The accumulation of large internal fat stores associated with long and deep torpor bouts (as long as 15 days), and sometimes social thermoregulation, enable fat-storing animals to fast throughout winter. By contrast, food-storing species store large amounts of food in a burrow. These species are mainly granivorous, because seeds are the only food items that can be stored several months without rotting (Humphries et al., 2003b). Only a few species are food-storing hibernators, mainly hamsters and chipmunks and individuals typically weigh between 10 and 300 g and are solitary. During hibernation, food-storing animals undergo shorter torpor bouts than fat-storing species, but have longer interbout euthermic episodes during which individuals consume their food hoards (Wollnik and Schmidt, 1995; Humphries et al., 2001; Table 1).

**TABLE 1:** Hibernation parameters of the common hamster (*Cricetus cricetus*), a food-storing species, and the garden dormouse (*Eliomys quercinus*), a fat-storing species, under controlled conditions of ambient temperature (T_a_) and photoperiod.

**Hibernation parameters**	**Food-storing**	**Fat-storing**
		
	**Common hamster**	**Garden dormouse**
Hibernation duration (h)	2385.6 ± 494.4**	3908.1 ± 32.6
Number of torpor bouts*	26.6 ± 0.6	19.4 ± 2.4
Mean duration of torpor bouts* (h)	90.5 ± 2.1	195.1 ± 21.4
Mean duration of IBE* (h)	29.4 ± 1.9	5.5 ± 1.0
Minimal body temperature (°C)	0.97***	6.0 ± 0.8

Conditions were for common hamsters (except for minimal body temperature): T_a_ ∼ 8°C, LD 10/14 h; and for garden dormice: T_a_ ∼ 5.3°C, LD 0/24 h, i.e., constant darkness. Results are presented as mean values ± SE. Data originated from Mahlert et al. (2018) for the garden dormouse, and from Waßmer and Wollnik (1997) (**) and Wassmer (2004) (***) for the common hamster. *Body temperature threshold for torpor bouts or phases of interbout euthermia (IBE) is of 32°C for common hamsters or 18°C for garden dormice.

#### Metabolic Changes in Fat-Storing Hibernating Species According to Body Fat Mass Changes and Activity

**FIGURE 1:**
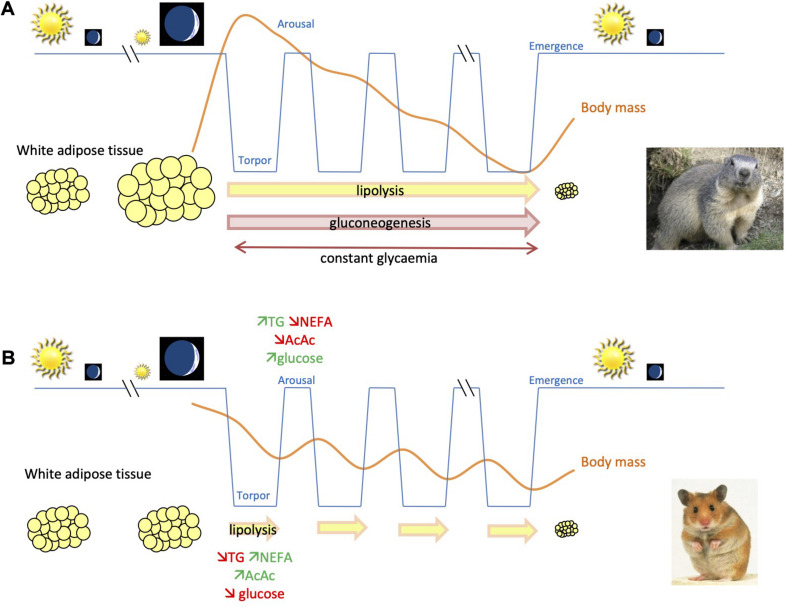
Schematic representation for comparison of body mass changes and use of energy substrates throughout hibernation in fat-storing and food-storing species. **(A)** In fat-storing hibernators, e.g., marmots, animals undergo a long-term fast during winter by mobilizing mainly lipids, accumulated prior to winter in white adipose tissue, to sustain their energy requirements. This leads to a substantial reduction of body (fat) mass of individuals over their winter hibernation fast. Energy can also come from gluconeogenesis, i.e., the synthesis of glucose from non-carbohydrate substrates during hibernation. **(B)** In contrast, food-storing hibernators, e.g., Syrian hamsters, experience a state of intermittent fasting during winter hibernation. Animals mobilize lipid stores during the torpor phases, but feed during interbout arousals, using glucose as main source of energy, and partially restore their body lipid/glycogen stores. In contrast to fat-storing species, glycemia decreases during torpor, which could constitute an endogenous signal for food-storing hibernators to arise from torpor and feed during interbout euthermia.

Fat-storing species show an increase in body (fat) mass prior to hibernation (Figures 1A, 3 left panel). Since individuals do not feed during the whole hibernation period, fat-storing hibernators rely on their fat stores to cover their energy requirements during winter. Plasma profiles of these animals during hibernation are characterized by a decrease in triglycerides, whereas plasma levels of ketone bodies increase (Rauch and Behrisch, 1981; Krilowicz, 1985; Nizielski et al., 1989). Lipid use and, more precisely, oxidation of non-esterified fatty acids (NEFAs) during hibernation are also supported by a respiratory quotient (RQ) close to 0.7 during torpor episodes (Lyman and Chatfield, 1955; Mokrasch et al., 1960; Snapp and Heller, 1981). Glycemia is maintained throughout hibernation thanks to the activation of gluconeogenic pathways (Twente and Twente, 1967; Tashima et al., 1970; Krilowicz, 1985; Florant et al., 1986; Nizielski et al., 1989). All these findings were recently confirmed via RNA sequencing and proteomic analyses in hibernating 13-lined ground squirrels (Hampton et al., 2011, 2013; Vermillion et al., 2015).

In fat-storing hibernators, the tissue showing the greatest changes during hibernation is probably the white adipose tissue (WAT). Therefore, it was interesting to look at adipokine levels over the seasons. Leptin is secreted proportionally to the amount of WAT. Hence, not surprising, plasma leptin levels are substantially high after fat accumulation, just before hibernation, compared to summer levels (*Marmota monax*: Concannon et al., 2001; *M. flaviventris:* Florant et al., 2004; *Dromiciops gliroides*: Franco et al., 2017; Table 2). However, this hormone is known to increase energy expenditure and to inhibit the initiation of torpor (Freeman et al., 2004). In the woodchuck, a large fat-storing species, it seems that plasma leptin levels and hypothalamic sensitivity to this hormone are uncoupled during the preparatory fattening phase prior to the hibernation onset (Concannon et al., 2001). In small hibernating species, uncoupling also exists, but between adiposity and plasma leptin levels, as shown in the little brown bat (*Myotis luciferus*: Kronfeld-Schor et al., 2000). Similar patterns have been observed in golden-mantled ground squirrels (*C. lateralis*: Healy et al., 2008) and arctic ground squirrels (*Urocitellus parryii:* Ormseth et al., 1996; Boyer et al., 1997). Concerning adiponectin, an adipokine that is secreted inversely proportional to lipid stores (Lafontan and Viguerie, 2006), only one study has assessed its variations during the annual cycle of a hibernating species (Florant et al., 2004). This study conducted in marmots (*M. flaviventris*) showed that adiponectin is low at the beginning of fat accumulation and elevated during hibernation (Florant et al., 2004). This seems consistent with the role of adiponectin in stimulating fatty acid oxidation (Fruebis et al., 2001; Masaki et al., 2003). Other hormones controlling metabolic pathways have been measured during hibernation compared to the active season in fat-storing species. Pancreatic hormone levels are low during hibernation in deep torpor, but increase during the rewarming phase (*Erinaceus europaeus*: Hoo-Paris and Sutter, 1980; Hoo-Paris et al., 1982; *Glis glis*: Castex et al., 1984; Hoo-Paris et al., 1985; *C. lateralis:* Bauman et al., 1987). Glucagon level remains high during arousals whereas insulin level decreases, probably to sustain glycemia during this phase of euthermia (Hoo-Paris et al., 1985). In larger species, pancreatic hormone levels remain low throughout hibernation (*M. flaviventris*: Florant et al., 1986). The orexigenic hormone ghrelin increases before hibernation and may stimulate food ingestion during this period of fat accumulation (*Citellus lateralis*: Healy et al., 2010). Plasma ghrelin concentration is low during hibernation in deep torpor and increases again upon arousal from torpor, as generally observed during stages of starvation (Cummings, 2006). Cortisol decreases during deep torpor and increases again during arousal phases, but stays lower than during the active foraging phase (Popova and Koryakina, 1981; *Citellus citellus*: Shivatcheva et al., 1988). This might prevent the induction of catabolic pathways, especially of body proteins, during hibernation.

**TABLE 2:** Plasma hormone concentrations (means ± SEM) of fat-storing species during long photoperiod (“LP”), short photoperiod (“SP”) hibernation at temperate (“SP warm”) or low (“SP cold”) ambient temperatures.

**Hormones**	**LP**	**SP warm**	**SP cold**			**Species**	**Photoperiod/temperature**	**References**
			
			**Torpor**	**Re-warm**	**Arousal**			
Insulin (pg.mL^–1^)	–	–	2.26 ± 0.38^a^	12.7 ± 3.5^b^	3.2 ± 0.9^a^	*Glis glis*	LP LD natural/SP LD_9:17 (5°C)	Castex et al., 1984
	–	–	0.73 ± 0.14^a^	0.90 ± 0.10^a^	0.73 ± 0.10^a^	*Marmota flaviventris*	SP LD_8:16 (5 ± 2°C)	Florant et al., 1986
Glucagon (pg.mL^–1^)	–	–	206 ± 14^a^	334 ± 36^b^	268 ± 21^b^	*Glis glis*	LP LD natural/SP LD_8:16 (5°C)	Hoo-Paris et al., 1985
	–	–	82 ± 9^a^	75 ± 8^a^	94 ± 6^a^	*Marmota flaviventris*	SP LD_8:16 (5 ± 2°C)	Florant et al., 1986
Adiponectin (AU)	1.2	1.2	–	–	–	*Marmota flaviventris*	LP LD natural (20 ± 3°C)/SP LD_0:24 (5°C)	Florant et al., 2004
Leptin (ng.mL^–1^)	3.2^a^	–	10.7^b^			*Marmota flaviventris*	LP LD natural (20 ± 3°C)/SP LD_0:24 (5°C)	Florant et al., 2004
Ghrelin (ng.mL^–1^)	3.6^a5.6^^*b*#x2013;^	∼3^a^	–	∼4^b^	*Citellus lateralis*	LD natural (20 ± 2°C)/SP LD_0:24 (5°C)	Healy et al., 2010
Cortisol (ng.mL^–1^)	77.3 ± 20.3	51.2 ± 3.8	19.2 ± 0.5^a^	–	36.2 ± 2.2^b^	*Citellus citellus*	LP LD natural (16-26°C)/SP LD_0:24 (7 ± 1°C)	Shivatcheva et al., 1988

*Different superscripts indicate significant differences (*p* < 0.05) between columns from the same raw. Adapted from Weitten et al. (2013).*

#### Metabolic Changes in Food-Storing Hibernating Species

**FIGURE 2:**
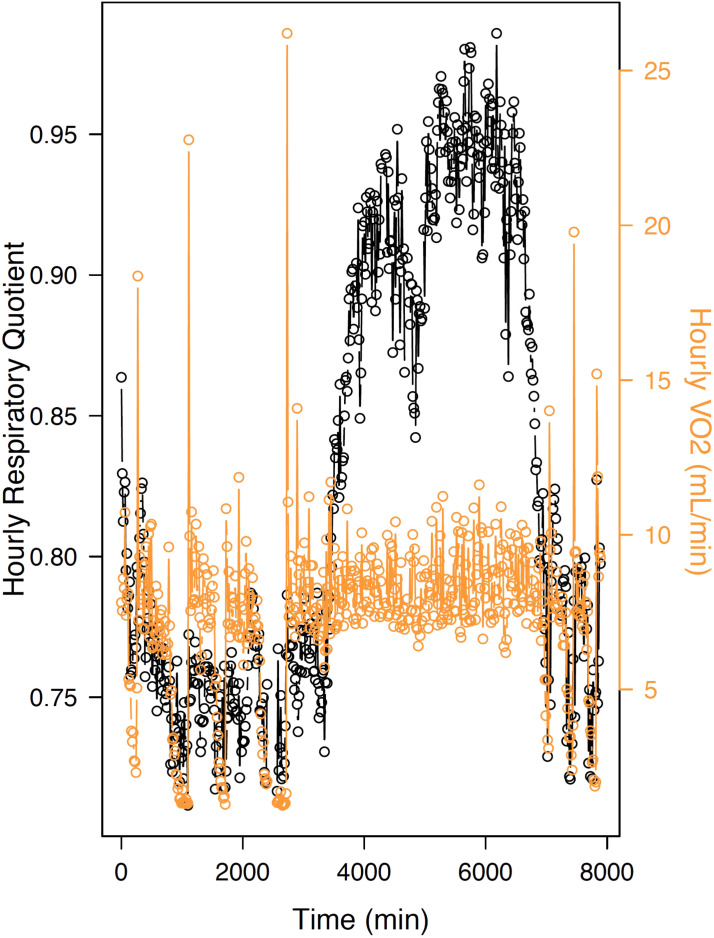
Hourly variations in MR (“VO_2_”) and respiratory quotient (“RQ”) toward the end of hibernation and after emergence in the common hamster, a food-storing hibernator. During torpor, VO_2_ decreases substantially and RQ drops to values close to 0.7, indicating the primary oxidation of lipid to fuel energy requirements at low temperatures. Upon arousals, RQ increases to intermittent values or ∼0.8 (indicating oxidation of mixed substrates), whereas values rise to ∼0.9 at emergence from hibernation and further (0.95) toward the active season. The arrow represents the termination of hibernation, indicating an almost exclusive metabolization of carbohydrates.

Contrary to fat-storing hibernators, food-storing species do not gain weight before hibernation, or if they do, to a much lesser extent than fat-storing species. However, food-storing hibernators consume body fat (retroperitoneal and peri-epididymal fat pads) during hibernating bouts, as indicated by RQ close to 0.7 in torpor (Figure 2), and a decrease in triglyceridaemia, whereas plasma NEFA concentration increases (*Mesocricetus auratus*: Weitten et al., 2013). These NEFAs are mainly converted into acetoacetate. In contrast to fat-storing species, glycemia decreases during deep torpor, which could constitute an endogenous signal for food-storing hibernators to arise from torpor and feed during interbout euthermia. During arousals, the plasma profile of hamsters is characterized by a post-prandial state, with an increase in glycemia and triglyceridaemia and a decrease in plasma concentrations of both NEFAs and ketone bodies (Figure 1B). As described above, in fat-storing species, most hormones are at their nadir during hibernating bouts. This is also the case for food-storing species (Table 3). As an example, pancreatic hormone levels are low during deep torpor in the Syrian hamster (*M. auratus*) during hibernation but also during the pre-hibernation phase and upon arousal (Weitten et al., 2013). This is surprising because these animals are consuming stored food during arousal phases, leading to changes in plasma metabolite levels and especially glycemia that should trigger changes in pancreatic hormone secretion. However, activity of the pancreas seems to remain low in food-storing species under short photoperiod conditions, which is known to induce a winter-like phenotype in the individuals. The gut regulatory peptides GLP-1 and GIP also decrease significantly under short photoperiod (non-hibernating) conditions and during hibernation in deep torpor, but show a slight increase after food ingestion during arousal phases (Weitten et al., 2013). In fact, these peptides are generally secreted in response to food ingestion to enhance anabolic pathways (Baggio and Drucker, 2007). In food-storing species, a decrease in corticosteronemia during torpor probably prevents body protein catabolism, like in fat-storing species (Weitten et al., 2013). This latter hypothesis may be supported by an absence of increase in uremia during torpor. As in small fat-storing species, leptinemia decreases in the pre-hibernation phase (Weitten et al., 2013), suggesting an uncoupling between adiposity and leptin secretion, probably to induce a torpid state. Also, as in fat-storing animals, plasma adiponectin levels remain high throughout hibernation (Weitten et al., 2013), probably to stimulate NEFA oxidation. Adiponectin is also known to play a role in thermogenesis (Masaki et al., 2003) and could therefore enable food-storing species to maintain T_b_ at a few degrees above ambient temperature (T_a_) during hibernation in deep torpor.

**TABLE 3:** Plasma hormone concentrations (means ± SEM) of Syrian hamsters (*Mesocricetus auratus*) under long photoperiod at 20°C (“LP20”), short photoperiod at 20°C (“SP20”), and short photoperiod at 8°C in torpor (“Torpor”) or during arousal phases (“Arousal”).

**Hormones**	**LP20**	**SP20**	**Torpor**	**Arousal**
Insulin (ng.mL^–1^)	1.60 ± 0.34^a^	0.44 ± 0.06^b^	0.86 ± 0.08^ab^	0.39 ± 0.04^b^
Glucagon (pg.mL^–1^)	238.4 ± 62.3^a^	51.8 ± 23.2^b^	53.2 ± 13.7^ab^	38.2 ± 12.0^b^
GLP-1 (pg.mL^–1^)	108.53 ± 24.50^a^	6.31 ± 3.25^b^	2.24 ± 0.26^b^	14.39 ± 3.49^bc^
GIP (pg.mL^–1^)	21.79 ± 4.90^a^	6.62 ± 2.09^a^	5.35 ± 1.03^a^	13.09 ± 5.15^a^
Leptin (ng.mL^–1^)	1.61 ± 0.10^a^	0.58 ± 0.02^b^	0.80 ± 0.09^ab^	0.78 ± 0.06^ab^
Adiponectin (ng.mL^–1^)	89.4 ± 2.3^a^	95.1 ± 3.8^a^	125.6 ± 6.6^a^	75.9 ± 2.0^a^
Corticosterone (ng.mL^–1^)	5.93 ± 0.45^ab^	9.23 ± 0.42^a^	1.00 ± 0.06^b^	8.39 ± 0.67^a^

*Different superscripts indicate significant differences (*p* < 0.05) between photoperiodic and physiological states. Adapted from Weitten et al. (2013).*

#### Gastro-Intestinal Adaptations of Food-Storing Species

Complete atrophy of the intestinal mucosa occurs in fat-storing species during hibernation (*Ictidomys tridecemlineatus:* Carey, 1990, 1995); *Marmota marmota*: Hume et al., 2002). In food-storing species, however, intestinal morphology is preserved and jejunal villi length even increases during torpor, as described in Syrian hamsters (*M. auratus*; Weitten et al., 2013). Despite mucosal atrophy during hibernation, fat-storing species show a maintenance of the expression of some intestinal enzymes and transporters such as those involved in glucose absorption (Carey and Martin, 1996). Similarly, Galluser et al. (1988) observed that enzymes involved in protein and sugar hydrolysis were expressed throughout hibernation in the common hamster (*Cricetus cricetus*), a food-storing species. A further study of common hamsters even showed that intestinal hydrolysis of triglycerides, starch, and protein was maintained during hibernation as well as NEFA and glucose absorption (Weitten et al., 2016). Glucose absorption was even increased in hibernating hamsters, certainly to restore glycemia and glycogen stores for the subsequent hibernating bout. In another food-storing species, the eastern chipmunk (*Tamias striatus*), digestive efficiency was positively correlated to torpor use (Humphries et al., 2001). According to these authors, food-storing species could acquire a twofold advantage in undergoing a torpid state: energy sparing and increased efficiency for assimilating fuel reserves.

#### Biomedical and Evolutionary Implications of Contrasted Hibernation Strategies: The Case of Fat- vs. Food-Storing Hibernators

One can consider hibernation in fat-storing species as a continuous state of long-term fasting, whereas food-storing hibernators alternate short fasting and feeding bouts, which resembles intermittent fasting. These two types of fasting, i.e., continuous and intermittent, could have major biomedical implications in terms of depression of MR and positive effects of fasting on human health and longevity, as highlighted by a number of studies in this field (for reviews, see Martin et al., 2006; Golbidi et al., 2017; Patterson and Sears, 2017; de Cabo and Mattson, 2019). Indeed, these contrasted responses to metabolic depression and intermittent or long-term fully reduced calorie intake are transduced into specific metabolic adaptations, including the maintenance of a fully-functional digestive tract in food-storing species throughout the hibernating season. Such a strategy should lead to a more positive energy balance in these individuals during hibernation compared to fat-storing species.

Beyond biomedical considerations, what do these contrasted hibernation behaviors imply in terms of evolutionary perspectives? This can be addressed by comparing different life-history traits between fat- and food-storing hibernators. At first, overwintering survival was assumed to be worse in food-storing species because of a risk of rotting or pilfering of food hoards (Davis, 1976; Vander Wall, 1990). However, food-storing animals should have a better body condition upon emergence from hibernation, due to their ability to assimilate food along winter hibernation (Humphries et al., 2001). Therefore, food-storing animals can immediately mate after hibernation, and are able to produce more litters per year compared to fat-storing species. These litters are generally composed of more offspring that grow more rapidly and become fertile earlier (sometimes within their year of birth), leading to a better reproductive success in food-storing than in fat-storing species. However, the maximum lifespan is higher in fat-storing hibernators than in food storing species or non-heterotherms (Wolf et al., 2007; Careau et al., 2009; Turbill et al., 2011; Ruf et al., 2012). Hence, from an evolutionary perspective, the fat-storing strategy might correspond to a slow life history, with a trade-off in favor of survival, whereas the food-storing strategy might be in line with a fast life history, with short life expectancy and a trade-off in favor of reproduction.

### Torpor in the Tropics – Opportunistic (Daily) Torpor vs. Seasonal Hibernation

Another case of contrasted use of heterothermy and its ecological and evolutionary implications is highlighted by adaptive mechanisms occurring in opportunistic and seasonal heterotherms. In particular, comparisons of the gray mouse lemur (*Microcebus murinus*) and the “monito del monte” (*D. gliroides*) are expected to bring new insights on the metabolic origins of hibernation among tropical and sub-tropical species, and notably based on molecular aspects supporting the view of the existence of a continuum of hypometabolic responses between daily torpor and hibernation.

#### Two Cases of Opportunistic Heterotherms: Lemurs and Monitos

In this section, we will refer to “hibernation” as a synonym for “seasonal obligatory long-lasting torpor bouts,” occurring in a predictable manner, to distinguish it from “daily opportunistic torpor,” which occurs among species that perform short (few hours) and shallow torpor events (T_b_ reduction to 15–18°C) (Song and Geiser, 1997; Bartels et al., 1998; Bozinovic et al., 2007), and generally occurs as an adaptation to unpredictable environments (Ruf and Geiser, 2015; Geiser, 2020). These authors maintain that the evolutionary origin and functional meaning of daily opportunistic torpor is different from that of seasonal torpor, and they classify species separately between “daily heterotherms” or “seasonal heterotherms,” but not without debate (Boyles et al., 2013; Nowack et al., 2020). With this criterion, more than 50% of Australian mammal species appear to exhibit some form of heterothermy, showing great physiological diversity, ranging from the long hibernation bouts of echidnas (7 months) to the short torpor bouts of Dasyurid marsupials and bats (Geiser and Körtner, 2010). African heterotherms include Madagascar tenrecs (*Echinops telfari*) that display seasonal torpor (Lovegrove and Genin, 2008), elephant shrews (*Elephantulus*) with random patterns of hibernation-daily torpor (Lovegrove et al., 2001), Cheirogaleidae lemurs (Storey, 2015; Faherty et al., 2016) that show daily torpor even at “warm” temperatures, and the mouse lemur (*M. murinus*) that shows flexible heterothermic phenotypes (Schmid and Ganzhorn, 2009). Daily torpor has been described for a few South American species including hummingbirds (Carpenter, 1974; Wolf et al., 2020), bats (Bozinovic et al., 1985), rodents (Bozinovic and Marquet, 1991), and didelphid marsupials (Bozinovic et al., 2007; Geiser and Martin, 2013). However, seasonal torpor is only known in the Microbiotherian “monito del monte,” *D. gliroides* (Bozinovic et al., 2004).

#### Monito del Monte, a Flexible Hibernator

**FIGURE 3:**
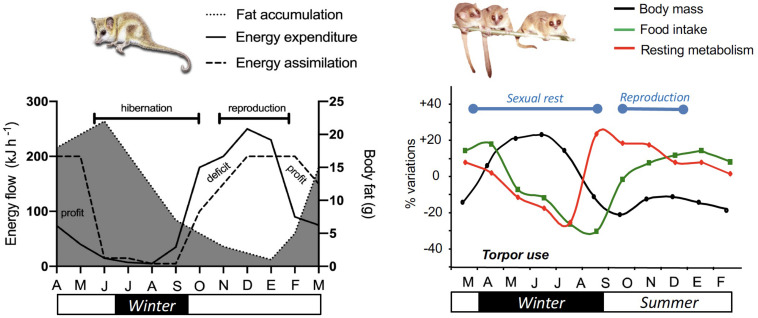
Hypothetical (but realistic) annual budget of energy and activity in monitos and mouse lemurs. Curves for South American monito del monte (*Dromiciops gliroides*) (left panel) are based on several descriptions of the reproductive cycle (Muñoz-Pedreros et al., 2005), seasonal variations in activity, adiposity and body mass (Celis-Diez et al., 2012; Franco et al., 2017) and food availability (Franco et al., 2011; di Virgilio et al., 2014). After reproduction in March (end of summer), monitos start to reduce activity and energy expenditure, and accumulate almost twice their body size in fat (Franco et al., 2017). The net annual energy balance is usually close to zero and varies depending on cold winter temperatures, allowing torpor to occur. Annual variations of body mass, MR (Perret, 1997) and food intake in captive male mouse lemurs (*Microcebus murinus*) native to Madagascar (right panel) show drastic changes across seasons, similar to that observed in wild conditions. At the beginning of winter, male mouse lemurs make extensive use of daily torpor to save energy and therefore fatten to increase their body mass by ∼40%. They reach a plateau in the middle of winter and spontaneously increase their MR while food intake is still low. Such changes coincide with the recrudescence of the reproductive axis, and lead to body mass loss. High MRs during and after mating season are supported by increased foraging, thus leading to stable body mass throughout the summer.

**FIGURE 4:**
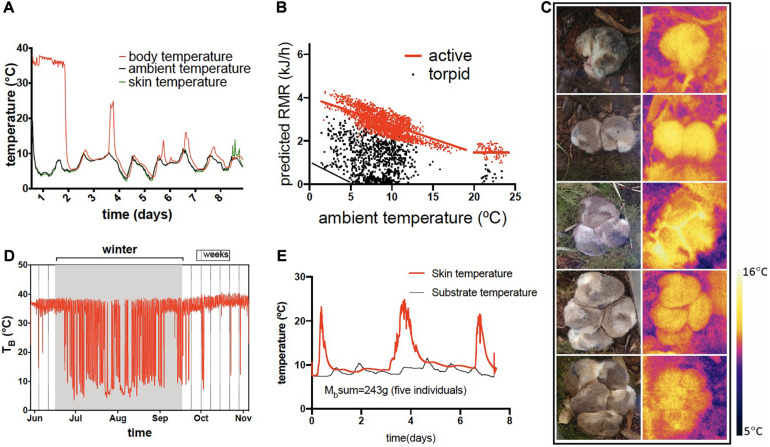
Flexible hibernation in *Dromiciops gliroides*. **(A)** a single torpor bout of 8 days in one individual; **(B)** predicted resting metabolic rate (RMR) in one individual followed during one winter, using the formula RMR = c(T_b_–T_a_); from an intraperitoneal data logger) where c = minimum thermal conductance for *D. gliroides* (c = 3.49 J g^–1^h^–1^°C^–1^), from Bozinovic et al. (2004) showing the frequent torpor episodes at relatively warm temperature except when reaching the critical T_A_ = 5°C, when thermoregulation in torpor begins (black line); **(C)** social hibernation showing animals in torpor and thermographic images of the groups; **(D)** body temperatures in a single individual during one hibernating cycle in the field showing the frequent arousals (this animal received supplementary food) and, **(E)** skin temperature (red) of a cluster of five individuals that were in torpor during 3 weeks (no food available); this plot shows the last 8 days (animals remained in the same site during these normothermic episodes).

The “monito del monte” (“monitos,” hereafter) is a hibernating South American marsupial (Bozinovic et al., 2004) phylogenetically more related to Australian marsupials than to other South and North American marsupial species (Mitchell et al., 2014). This species is distributed in a latitudinal range of about 1000 km, closely associated with the temperate rainforests of Southern South America (“Valdivian” forests) in Chile and Argentina and covering altitudes ranging from 200 m.a.s.l. in coastal forests to 1600 m.a.s.l. in high Andean *Nothofagus pumilio* (“lenga”) forests (Martin, 2010; Valladares-Gómez et al., 2019). Hibernation in monitos extends from approximately May to September and animals show a marked seasonality in fat deposits and energy expenditure (Figure 3). They are strictly arboreal (Godoy-Güinao et al., 2018), social and omnivorous with great preference for fruits which make them important dispersers of several native plants (Amico and Aizen, 2000; Amico et al., 2009). Monitos hibernate in clusters of four to nine individuals in elaborate nests that they build using mosses and South American bamboo (*Chusquea quila*); these potentially represent a strategy for heat conservation in winter (Celis-Diez et al., 2012; Nespolo et al., 2020). Although initially described as seasonal heterotherms (Bozinovic et al., 2004), recent field studies using miniature data loggers for simultaneously recordings of T_a_ and T_b_ over the whole hibernation cycle, revealed that monitos can experience long torpor bouts in winter (Figure 4A), but can also undergo short daily torpor episodes in any time of year, even at mild temperatures (T_a_ = 20°C, see Nespolo et al., 2010). While in deep torpor, monitos defend a critical T_b_ of about 5°C and thermoregulate in torpor (Figure 4B, black line). Interestingly, animals show the typical endothermic increase in MR when active and exposed to low T_a_ (Figure 4B, red line), but at the same time can experience torpor bouts at any T_a_ above the critical defended temperature. Monitos cluster together in hibernacula, which permit them to limit heat loss (Figure 4C) but, when food supplemented, individuals experience frequent arousals in winter (Figure 4D). At the same time, animals can experience long torpor episodes of up to 31 days without feeding and showing collective arousals and euthermic periods of 24–48 h (Figure 4E). Thus, monitos represent a case of a species experiencing both daily torpor and hibernation, and that modulate the use of heterothermy on a day-to-day basis depending on food supply (Nespolo et al., 2020). This flexible hibernator has been one of the best studied marsupial models of heterothermy from the molecular point of view.

#### Monitos as a Model of Marsupial Torpor

Although hundreds of studies have described the physiological underpinnings of heterothermy in marsupials, especially for Australian species (summarized in Geiser and Körtner, 2010), the molecular biology and biochemistry of marsupial heterothermy has been particularly well described in monitos, a South American marsupial. Monitos trigger their heterothermic state primarily due to a drop in T_a_, in food-deprived individuals with photoperiod and fat stores also being important modulators of torpor susceptibility (Nespolo et al., 2010, 2020). The transition from endothermy to heterothermy is characterized by passive cooling after a sudden reduction in MR, to a limit of about 1–2°C, when monitos start defending T_b_ by active thermoregulation (Figure 4B). It is highly likely that monitos hibernate at freezing temperatures, since several populations in Chile and Argentina are distributed in Andean locations where temperatures go below zero in winter (Balazote-Oliver et al., 2017; Valladares-Gómez et al., 2019). During torpor in monitos, usually T_b_ remains one or two degrees above T_a_, and animals could spend several weeks in this condition, where MRs are of about 5% of normothermic values, heart frequency can reach 3 beats per minute and breathing frequency could be less than 1 per minute (Nespolo et al., 2010). Deep torpor is common in winter, but monitos often experience daily torpor in summer and spring (Figure 4C).

#### The Gray Mouse Lemur, a Primate Model of Flexible Heterothermy

**FIGURE 5:**
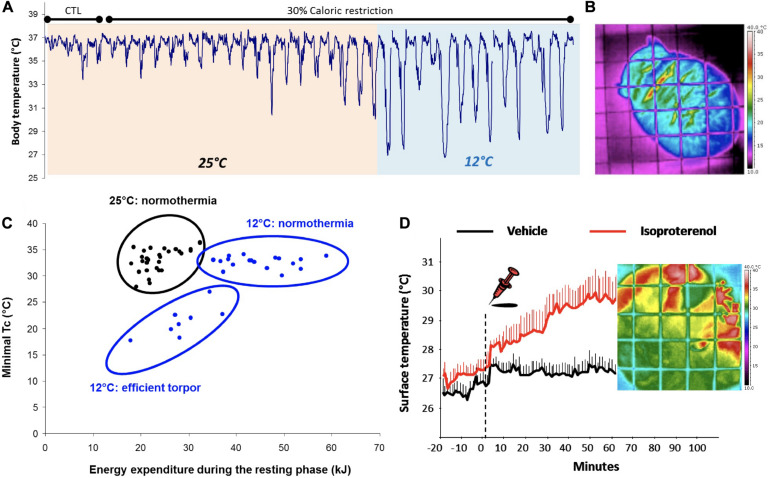
Flexibility of heterothermy in captive *Microcebus murinus.* **(A)** Captive mouse lemurs adjust the depth and duration of torpor bouts depending on food supply (control CTL vs. 30% caloric restriction CR) and ambient temperature (25 vs. 12°C); **(B)** Example of thermographic image of a mouse lemur acclimated to 12°C; the surface temperature is maintained very low, witnessing the insulating capacity of the animal, except at the inter-capsular region where brown adipose tissue is present; **(C)** Graph showing the relationship between minimal core temperature and energy expenditure during the resting phase recorded in animals acclimated to 25 and 12°C; the data clearly demonstrate the existence of an inter-individual variability in the response to cold (either maintenance of normothermia or deep torpor bouts); **(D)** Data showing the rise of surface temperature (heat dissipation mostly through hands and feet) following the injection of isoproterenol, a chemical that stimulates non-shivering thermogenesis.

The gray mouse lemur (*M. murinus*; Strepsirrhini; Lemuriformes; Cheirogaleidae) is a small nocturnal primate species (body mass: 60–90 g) originating from Madagascar. Half-life expectancy is around 5.7 years but animals can live for 8–10 years in captivity (Perret, 1997). The species as been widely used as a model for aging studies (Languille et al., 2012) and exhibits robust changes in body composition that are synchronized on photoperiodic cycles, a feature that is conserved in captivity (Perret and Aujard, 2001). These variations in body composition result from deep changes in physiological characteristics during winter, in particular the use of hypometabolism, even in captivity (Figure 5). Mouse lemurs adjust the depth and duration of torpor bouts according to feeding status and T_a_ (Figures 5A,B), and show an interesting inter-variability in their response (Figure 5C). Recent work (Terrien et al., 2017) has shown that captive male mouse lemurs acclimated to winter-like photoperiod (10 h/day) for 6 months and maintained in constant favorable housing conditions exhibit large changes in body mass explained by variations in metabolic levels and food intake. Such changes result in a biphasic phenotype: a phase of massive body mass gain followed by the spontaneous arrest of fattening. Strikingly, mouse lemurs are protected against glucose intolerance during fattening, but not during the second half of winter when they lose fat and exhibit increased fasting insulin levels. Most importantly, these natural transitions are fully reversible, and never reach pathological levels, as reflected by the apparent absence of an inflammatory response (Terrien et al., 2017). The second half of winter corresponds (only for male mouse lemurs) to the recrudescence of the reproductive system, which is completely regressed during the first half of winter (Perret and Aujard, 2001). This regression/recruitment phenotype can be interpreted as a yearly puberty that could repeatedly occur in both males and females (read below), year after year.

Although generally considered to be a daily heterotherm, records of hibernation episodes (up to 30 days) have been reported in wild female gray mouse lemurs (Schmid and Ganzhorn, 2009). There seems to have a strong inter-individual variability in this feature, probably due to differences in habitat structure (habitat fragmentation), nutritional state (food availability), and body composition (fat stores). Torpor is triggered to save energy and water (Humphries et al., 2003b) but is somehow also a costly process, as highlighted by recent work on telomere dynamics during hibernation (Giroud et al., 2014; Hoelzl et al., 2016; Nowack et al., 2019). Although supported by behavioral strategies (Terrien et al., 2011), arousals from torpor are very demanding as they require the activation of NST in BAT. The activation of such process induces rapid and efficient production of body heat (Figure 5D), but also consumes fat reserves. The balance between the energy saved during torpor vs. the energetic costs of arousal is known to be altered by the aging process. Indeed, aged mouse lemurs display deficiencies in controlling the drop in T_b_ when exposed to low T_a_ (Terrien et al., 2008), thereby necessitating higher energy input for subsequent rewarming (Terrien et al., 2009). This leads to a faster exhaustion of fuel reserves, notably in BAT (Terrien et al., 2010a). These results are further supported by the enhanced use of behavioral adjustments during aging in this species (Terrien et al., 2010b) as a compensation for age-related deficiencies in physiological responses to cold exposure.

An additional parameter, quite neglected in the literature, that introduces variability in the heterothermic response is sex (Vuarin et al., 2015). Indeed, hibernation episodes during the dry season have only been reported in females but not males (Schmid and Kappeler, 1998; Schmid, 1999), supporting the “female thrifty hypothesis” that confers to females an enhanced capacity to display hypometabolism (Jonasson and Willis, 2011). Such sex bias could be linked to the phenological shift that exists between males and females in the yearly period during which they invest energy in reproduction. Female reproductive investment centers on gestation, lactation, and juvenile care and occurs during the wet favorable season. By contrast, male mouse lemur reproductive investment centers on male-to-male competition at the mating season (early summer) and, most importantly, on spermatogenesis that is a long-lasting process and has to start at the end of the dry season to ensure male readiness at the mating season. Such phenological differences between males and females might determine the flexibility of torpor bouts in mouse lemurs. There are a number of indications of enhanced torpor efficiency in females as compared to males. Indeed, although hibernation episodes have never been observed in captivity, captive female mouse lemurs are capable of using torpor during gestation and lactation when facing food shortage (Canale et al., 2012), whereas the male response to a comparable stress shows a relative inefficiency to maintain body mass by using torpor (Giroud et al., 2008; Villain et al., 2016). In addition, female mouse lemurs show enhanced mitochondrial and antioxidant capacities (Noiret et al., 2020).

#### Monitos and Mouse Lemurs: Daily and Seasonal Heterotherms in the Same Species

Torpor use in its depth and duration seems to be highly flexible in mouse lemurs and monitos and such flexibility probably requires specific physiological characteristics and are highly constrained by life-history traits and environmental conditions. For example, it is well referenced that hibernation capacities rely on the ability to store enough fat in order to sustain long periods of foraging and/or metabolic inactivity. As a consequence, such a phenotype is mostly expressed by larger heterotherms (Ruf and Geiser, 2015). However, among small heterotherms, including monitos and mouse lemurs, individuals showing the best body condition, i.e., the highest fat stores, also have the capacity to extend their periods of hibernation to several weeks. By contrast, inappropriate use of daily torpor or hibernation, i.e., when extrinsic (environmental) or intrinsic (body condition) factors are not favorable, might not bring the expected benefits of using hypometabolism. Indeed, a recent perspective paper proposes that physiological flexibility, including hypometabolic response to environmental constraints, not only offers ecological and physiological benefits, but also induces important costs, e.g., oxidative damage and immunodeficiency (Landes et al., 2020). Comparable observations of torpor flexibility have been made in lesser hedgehog tenrecs, *Echinops telfairi* (Lovegrove and Genin, 2008) and stripe-faced dunnarts (*Sminthopsis macroura*, Körtner and Geiser, 2009; for review see Nowack et al., 2020). Such physiological features, i.e., being able to either display short daily torpor bouts or longer weekly hibernating periods in the same species, contribute to the view that rather than being two distinct functional traits, hibernation, and daily torpor are probably extremes of a continuum (Boyles et al., 2013).

#### Daily Torpor and Hibernation as a Continuum of Hypometabolic Responses: Insights From Molecular Analyses

Based on observations from molecular analyses of the torpid state (for reviews, see Van Breukelen and Martin, 2002; Storey and Storey, 2004, 2007; Staples, 2011; van Breukelen and Martin, 2015), it appears that the daily expression of torpor and the successive use of multi-day or weeks-long torpor, also defined as hibernation, stand as two extremes along the continuum of hypometabolic responses. Indeed, a range of molecular experiments done with *D. gliroides* -a species expressing both daily torpor and seasonal hibernation- reveal that monitos experience immunological depression and muscle atrophy during torpor (Franco et al., 2013, 2017), whereas other analyses suggest the existence of tissue-specific responses regulating key molecular pathways by microRNAs to limit oxidative damage and muscle atrophy (Hadj-Moussa et al., 2016), and overexpressing cryoprotective proteins against ischemia/reperfusion stress (e.g., heat shock proteins) in the liver, heart, and brain (Nespolo et al., 2018). Interestingly, these authors found a single transcript encoding for the thioredoxin interacting protein (*TXNIP*) that was strongly overexpressed in several tissues, but especially in the brain. This is a potent antioxidant protein involved in regulating mitochondrial function to help suppress oxidative metabolism when oxygen is limiting and regulating a metabolic shift to anaerobic glucose catabolism by mediating inhibition of pyruvate dehydrogenase (Spindel et al., 2012; Chong et al., 2014; Yoshioka and Lee, 2014). In mice, the *TXNIP* gene is overexpressed in the hypothalamus, liver, and white and brown adipose during induced-torpor experiments as well as in natural torpor in Siberian hamsters, *Phodopus sungorus* (Hand et al., 2013; DeBalsi et al., 2014; Jastroch et al., 2016).

In the same vein, recent data show that some genes regulating daily and prolonged torpor in mouse lemurs, although altered in a much less extensive manner, are shared with that of classical hibernating species, but can also show some specificities. For example, miRNAs are known regulators of metabolic flexibility in hibernating mammals (Hadj-Moussa et al., 2016; Arfat et al., 2018; Biggar and Storey, 2018) and are particularly involved in growth processes. Such regulation of gene expression also seems to be involved in the torpor response of mouse lemurs (Biggar et al., 2018; Hadj-Moussa et al., 2020). In particular, mouse lemur torpor use involves miRNA inhibition of gene transcripts related to energetically-unfavorable cellular processes in order to facilitate metabolic suppression (Hadj-Moussa et al., 2020). For instance, small RNA sequencing of lemur miRNAs in skeletal muscle revealed that muscle-specific miR-1 and miR-133 are downregulated in mouse lemurs during daily torpor. These “myomiRs” are likely to have important roles in preventing disuse-induced muscle atrophy by targeting genes involved in regulating muscle development and energy use. For example, mir-1 and miR-133 are generally upregulated in skeletal muscles of warm-hibernating Swedish brown bears (Luu et al., 2020), the daily- or seasonally-torpid marsupial, monito del monte (Hadj-Moussa et al., 2016), and in the foot muscle of anoxia- and freeze-tolerant sea snails (Biggar et al., 2012), likely helping to protect their muscles from sustaining damage as they lie dormant. A decrease in these miRNAs in lemurs is significant and emphasizes the need to study molecular adaptations in a range of species capable of metabolic suppression, especially through the unbiased approach of sequencing all available small non-coding RNAs. Regulation of energy homeostasis through AMPK signaling, especially to promote ATP sparing and partitioning, also seems to be similar in the mouse lemur to that of classic hibernators (Zhang et al., 2015). Protein kinases involved in MAPK cascades also showed comparable regulation of expression and relative protein phosphorylation between mouse lemurs and hibernators (Biggar et al., 2015b). For instance, phosphorylation for ERK1/2 and MEK was strongly negatively regulated in skeletal muscle during torpor in the mouse lemur and in the heart of hibernating ground squirrels (Childers et al., 2019). In addition, suppression of the immune system during torpor was observed in the mouse lemur, in line with what was already known in hibernators (Franco et al., 2013; Tessier et al., 2015a). Epigenetic regulation is also involved since miR-222 was reduced during torpor in the mouse lemur as well during hibernation in ground squirrels (Wu et al., 2014). By contrast, it seems that the regulation of heat shock proteins or proteins involved in the antioxidant machinery do not change as much during daily torpor (Biggar et al., 2015a) as during hibernation (Carey et al., 1999; Morin and Storey, 2007). Furthermore, hepatic regulation of the phosphorylation state of the insulin receptor showed contrasting effects, increasing in torpid mouse lemurs (Tessier et al., 2015b), and therefore potentially reflecting an inhibition of gluconeogenesis although this process that is known to be sustained during hibernation (Green et al., 1984).

Taken together, all the data for these two small mammal models (mouse lemurs and monitos) point toward specific molecular and cellular mechanisms for the adjustment of metabolic processes and protective functions during the extreme hypometabolic down-state of torpor. These adaptive mechanisms seem to be conserved in evolution among diverse heterothermic species known to date. Taking inspiration from natural adaptations constitutes a necessity in the current world of ongoing climate change and spreading pathophysiological pandemics. Therefore, exploring these outstanding mechanisms occurring naturally in heterothermic models constitutes a unique opportunity to develop efficient responses, tools and treatments to address major environmental and health concerns. Specifically, the study of these mechanisms have potential for a better understanding of (i) protective responses against metabolic disorders such as obesity or sarcopenia (Cotton, 2016), (ii) the homeostasis of neuronal functions, e.g., the maintenance of hyper-phosphorylation of Tau proteins involved in resistance to neuro-degenerative diseases (Härtig et al., 2007; Luppi et al., 2019), and (iii) the underlying mechanisms for a state of hypothermia in humans, also called “synthetic torpor,” for therapeutic goals (Cerri, 2017) or space exploration (Choukèr et al., 2019). In the context of the latter, the study of protective mechanisms for the torpid brain is of particular interest as well as the implications for the gaseous molecule H_2_S involved in the potential control and maintenance of metabolic depression and protective mechanisms in the torpid state.

## Protective Mechanisms During the State of Metabolic Depression

### Mechanisms to Protect the Torpid Brain

**FIGURE 6:**
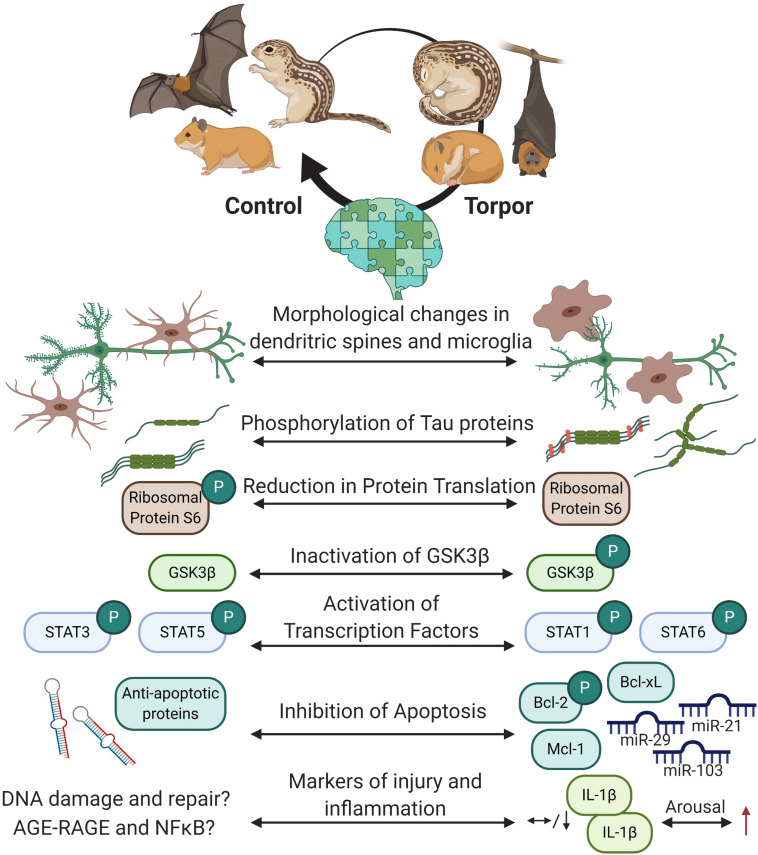
Overview of the protective mechanisms in the hibernating brain. Cells within the hibernating brain undergo rapidly reversible morphological changes and changes to structural proteins to prevent cellular damage. Energy expensive processes like protein translation are inhibited. Transcription factors like such as signal transduction and activators of transcription (STAT) are differentially phosphorylated but not by glycogen synthase kinase 3 beta (GSK3β) during torpor, and may regulate apoptosis via B cell lymphoma proteins (Bcl-2 and Bcl-xL) or myeloid leukemia cell differentiation protein (Mcl-1) protein expression and phosphorylation. Some microRNAs are also increased in the brain to prevent cell death. Markers of brain injury and inflammation have yet to be fully characterized, but current research shows that some cytokines that are low in the brain during torpor increase during arousal. Created with BioRender.com.

During torpor, hibernators may suppress their brain activity to the point where no electrical activity can be observed (Chatfield et al., 1951; Frerichs et al., 1994), but behind closed eyes, hibernating species coordinate the expression and activities of thousands of proteins (via changes in gene and protein expression, post-translational modification, and epigenetic controls) to ensure that one of the most important organs, the brain, is viable upon arousal (see Figure 6 for overview).

#### Regulation of Protein Translation and Tau Phosphorylation During Torpor

Most species suppress energetically expensive molecular processes during torpor to avoid metabolic disruption, when cellular resources of substrates to produce ATP may be limited. For example, protein translation is suppressed in brain of 13-lined ground squirrels (*Ictidomys tridecemlineatus*) to just 0.04% of euthermic levels (Frerichs et al., 1998) which was expected to correlate with a significant decrease in the relative expression levels of translated proteins during hibernation. However, multiple studies focusing on translational regulation in a range of hibernator tissues, including brain, indicate that hibernators use a much more rapid and reversible process to throttle translation: protein phosphorylation (Frerichs et al., 1998; Miyake et al., 2015; Logan et al., 2019). For example, ribosomal protein S6 is less phosphorylated at Ser235 during torpor, reducing its ability to bind the 5′-cap of mRNA and initiate translation (Miyake et al., 2015; Logan et al., 2019).

Consistent with these observations, hibernators including European ground squirrels (*Spermophilus citellus*), Arctic ground squirrels (*U. parryii*), black bears (*Ursus americanus*), and Syrian hamsters (*M. auratus*) use reversible phosphorylation of structural proteins such as Tau, to facilitate dendritic retraction and reduce neuronal cell signaling (Bullmann et al., 2016). Tau phosphorylation is a common feature of many tauopathies, which are neurodegenerative disorders associated with the build-up of proteinaceous plaques including tau, but fascinatingly, hibernators can reverse tau phosphorylation and clear protein aggregates upon arousal from deep torpor. Glycogen synthase kinase 3 beta (GSK3β) was postulated to regulate hibernator tau phosphorylation because it targets the specific phospho-sites that were differentially affected in hibernating Syrian hamster brain (Bullmann et al., 2016). However, new data from the Storey group indicates that GSK3β is inhibited during deep torpor in the forebrain and cerebellum of hibernating 13-lined ground squirrels (*Ictidomys tridecemlineatu*s), based on increases in p-GSK3β (S9) during torpor (Tessier et al., 2019). As a result, it is unlikely that GSK3β is part of a conserved mechanism governing hibernator tau phosphorylation across species.

#### Regulation of Inflammation and Apoptosis During Hibernation

In addition to their purported importance in reducing brain activity and maintaining brain functionality during hibernation, the neurons of cold-bodied hibernators pull their dendrites back into the cell bodies that sprouted them. This appears to be an effort to reduce tissue damage to the delicate, filamentous protrusions that are responsible for accepting information from the axons of other neurons and relaying those messages to the cell body (Popov et al., 1992). Microglia (neuronal immune cells) also change their morphology during torpor in hibernating hamsters such that their protrusions are thinned and appear disconnected from the main cell body (Cogut et al., 2018; León-Espinosa et al., 2018). Microglia have important roles in regulating dendrite elongation, neuronal plasticity and synaptic transmission of signals, in addition to producing inflammatory signaling molecules. Together, changes in dendrite and microglia morphology with a decrease in core T_b_ could serve to inhibit neuronal activity, and possibly inflammatory signaling during deep torpor. Hibernating animals may also facilitate changes in brain structure by differentially expressing genes involved in extracellular matrix plasticity and maintenance including collagens, laminins, integrins, and matrix metalloproteinases (Schwartz et al., 2013). Matrix metalloproteinases are also differentially expressed during torpor in hibernating hamster lung (Talaei et al., 2012), suggesting a role for tissue remodeling in the survival strategies of a range of hibernating species.

Interestingly, the enzyme GSK3β may regulate the DNA binding of transcription factors that suppress neuronal apoptosis and neuroinflammation. Specifically, GSK3β activation is required for control of signal transducer and activator of transcription (STAT) 3 and STAT5 activity but is not necessary for STAT1 or STAT6 activity in astrocytes (Beurel and Jope, 2008). Neither STAT3 nor STAT5 were differentially phosphorylated during hibernation in *I. tridecemlineatus* forebrain, cerebellum, or brainstem, suggesting that GSK3β inhibition during torpor could correlate with a decrease in the translocation of STAT3 and STAT5 to the nucleus (Tessier et al., 2019). By contrast, compared to winter euthermic and summer euthermic controls, respectively, forebrain and brainstem STAT1 was more phosphorylated during hibernation in deep torpor. In addition, both the cerebellum and brainstem of hibernating ground squirrels exhibit increased STAT6 phosphorylation levels at Y641 during deep torpor, compared with summer active and interbout aroused animals (Tessier et al., 2019). This transcription factor may play an important role in upregulating anti-apoptotic genes such as B-cell lymphoma 2 (Bcl-2) (Lee et al., 2018). Indeed, whole brain from hibernating ground squirrels shows a significant increase in the protein and phosphorylation levels of multiple anti-apoptotic proteins including Bcl-2, Bcl-xL, Bax-inhibitor 1, and Mcl-1 (Rouble et al., 2013). These molecular responses during torpor could help hibernating ground squirrels prevent brain damage during times of nutrient limitation.

Signal transducer and activator of transcription 6 has been implicated in mediating inflammation by facilitating macrophage recruitment, mucus production, and smooth muscle contractility, suggesting that its upregulation in the hibernating ground squirrel brain may help regulate neuroinflammation during natural torpor. Hibernators including hamsters and ground squirrels can mount inflammatory responses during deep torpor (Kurtz and Carey, 2007). At the molecular level, hibernators increase the local levels of pro- and anti-inflammatory cytokines (IL-1β, IL-4, IL-6, IL-10, TNFα, interferon γ) and potentially, leukocytes, based on increases in the relative levels of cell surface markers, in order to rapidly respond to exogenous and/or endogenous damage markers as they arouse from a hibernation bout (Kurtz and Carey, 2007; Cogut et al., 2018). Endogenous markers of damage can be sensed by receptors such as the receptor for advanced glycation end products (RAGE), that can then activate MAPK and JAK/STAT signaling cascades to increase the expression of pro-inflammatory transcription factors like NFκB. RAGE expression was recently discovered to be upregulated in hibernating ground squirrel adipose tissues, particularly as they enter or exit a hibernating bout (Logan and Storey, 2018). An increase in RAGE expression during entrance into deep torpor is consistent with a suppression of mitochondrial activity and therefore a potential for less efficient electron transport through the electron transport chain leading to ROS formation. Additionally, an increase in RAGE levels in arousing animals, when ROS levels increase as a result of increased breathing and MRs and elevated mitochondrial activity, suggest that RAGE is able to sense and respond to biomarkers of oxidative stress. Consistently, BAT in hibernating arctic ground squirrels shows increased levels of lipid peroxides and protein carbonyls during the rewarming period, and these oxidized lipids and proteins may be sensed by receptors such as RAGE to trigger their removal during interbout arousal (Orr et al., 2009). In addition, NFκB signaling is upregulated during deep torpor and arousal in ground squirrel cardiac muscle, skeletal muscle, and intestine, as well as hamster lung, which could contribute to increased pro-inflammatory gene expression (Carey et al., 2000; Allan and Storey, 2012; Talaei et al., 2012; Wei et al., 2018a). Interestingly, studies show that deep hibernators injected with inflammatory stimulants only respond to the exogenous markers of cell stress (e.g., *E*scherichia *coli* lipopolysaccharide) during interbout arousals, measured as an increase in T_b_ relative to saline-injected ground squirrels (Prendergast et al., 2002). By contrast, forebrain shows no signs of oxidized proteins (endogenous damage markers) during deep torpor, compared with euthermic controls, suggesting that brain may use molecular mechanisms such as elevated antioxidant capacity to prevent the accumulation of damaged proteins during hibernation (Orr et al., 2009). Other damage markers such as 8-hydroxy-2′-deoxyguanosine or markers of mitochondrial dysfunction have yet to be assessed in brain cells, in conjunction with an analysis of pro-inflammatory signaling cascades. However, an analysis of CD16–CD32 and CD68 expression as markers of microglia activity revealed that hibernating hamsters do not increase microglial activity (León-Espinosa et al., 2018). Though microglia do not display advanced morphological changes associated with microglial activation, the retraction of microglial dendrites and increases in microglial cell body to cell size ratio during deep torpor, as well as increases in the relative transcript levels of pro-inflammatory IL-1β and IL-6 upon arousal point to a role for microglia in the regulation of neuronal inflammation (Cogut et al., 2018). Together, these results suggest that hibernators may induce inflammation at particular time points of the torpor-arousal cycle, but much has yet to be explored with regard to the molecular mechanisms controlling hibernator neuroinflammation.

#### Other Brain Protective Mechanisms in Hibernation

Neuroprotection may also be facilitated through epigenetic mechanisms, including differential microRNA expression. In a study that focused on miRNAs that are commonly dysregulated in neurodegenerative conditions, hibernating little brown bats (*Myotis lucifugus*) showed significant increases in microRNAs during deep torpor that were involved in regulating focal adhesion, a process that involves changes in neuronal cell cytoskeletal structure, cell cycle progression, and cell death (Biggar and Storey, 2014). For example, miR-21, -29, and -103 were all significantly increased in brain of hibernating *M. lucifugus* and all have important roles in decreasing apoptosis (Roshan et al., 2014; Zhou and Zhang, 2014). These data, in conjunction with protein/phosphorylation data from *I. tridecemlineatus* studies and gene transcript data from hibernating greater horseshoe bats (*Rhinolophus ferrumequinum*) (Lei et al., 2014), suggest that hibernators may use conserved regulatory mechanisms acting at multiple levels (non-coding RNA, gene, protein, and post-translational) to limit neuronal cell death during hibernation in deep torpor.

### Role of H2S in Protective Mechanisms During Hibernation

Ever since the paper by Blackstone et al. (2005) that showed that inhalation of low concentrations of H_2_S confers a reversible metabolic suppression in mice, people have wondered whether H_2_S is involved in hibernation, for instance by inducing or maintaining torpor. Mechanistically, there are good reasons to explore this, since H_2_S can profoundly reduce mitochondrial respiration through inhibition of complex IV (cytochrome *c* oxidase) (Khan et al., 1990). Moreover, the past 15 years of H_2_S research has disclosed an array of protective effects that may be highly instrumental in hibernation to safeguard organ function and integrity in the face of hypoxia and/or excess reactive oxygen species production (Carey et al., 2000; Orr et al., 2009; Wu and Storey, 2014).

#### Endogenous H_2_S Production and Physiological Effects

**FIGURE 7:**
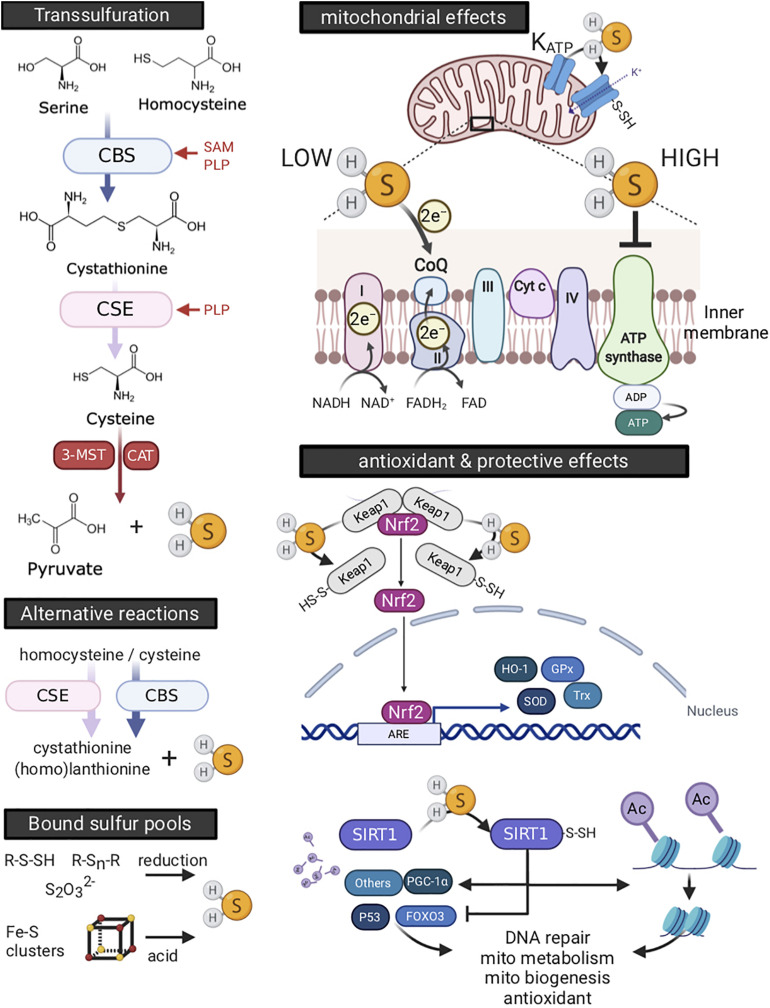
Endogenous production and physiology of hydrogen sulfide (H_2_S). H_2_S is enzymatically produced by the trans-sulfuration pathway or by alternative reactions, or derived from compounds binding sulfur (left side panels). H_2_S producing enzymes differ in various ways, including their dependence on co-factors, cellular, and tissue distribution. Optimal function for both cystathionine β-synthase (CBS) and cystathionine γ-lyase (CSE) is dependent on binding of pyridoxal-5′-phosphate (PLP, or vitamin B6) as a co-factor. In addition, S-adenosyl-L-methionine (SAM), the universal methyl group donor synthesized as an intermediate in the conversion of methionine to homocysteine, serves as a potent allosteric activator of CBS. The cellular localization of CBS and CSE is mainly cytosolic, yet they translocate to mitochondria under stressful conditions (Fu et al., 2012; Teng et al., 2013). 3-mercaptopyruvate sulfur transferase (3-MST) is located in both cytosol and mitochondria, with its activity increasing threefold in mitochondria (Nagahara et al., 1998). Endogenous H_2_S may also be derived non-enzymatically from “bound sulfur” pools. Upper compounds are sulfane sulfurs, such as thiosulfate (S_2_O_3_^2–^), persulfides (R-S-SH) and polysulfides (R-S_n_-R), which release H_2_S in the presence of reducing substances including glutathione (GSH) and cysteine (Ishigami et al., 2009) or by the action of 3-MST (Shibuya et al., 2009). Lower depicted compounds are acid-labile sulfides, which consist mainly of iron-sulfur clusters, of which the cubane-type [4Fe-4S] cluster is most common (Johnson et al., 2005). They are best known for their presence in proteins involved in oxidation-reduction reactions, including those of the electron transport chain in mitochondria. While degradation of iron-sulfur clusters releases H_2_S, this occurs at strong acidic conditions (pH < 5.4), likely limiting their contribution to endogenous H_2_S generation even under pathological conditions (Ishigami et al., 2009). H_2_S affects mitochondria (upper right panel) and produces antioxidant defense (lower right panel). Stimulation of oxygen consumption and ATP production occurs at low H_2_S concentration and originates from electron donation at coenzyme Q (CoQ) between complexes II and III (Goubern et al., 2007; Módis et al., 2013). At high concentrations, H_2_S competitively and reversibly inhibits cytochrome c oxidase (complex IV) (Khan et al., 1990; Módis et al., 2014). Moreover, high concentrations of H_2_S still donate electrons at coenzyme Q, which now reversely flow to complex II, reducing fumarate into succinate (Fu et al., 2012). Moreover, H_2_S activates the mitochondrial K_ATP_ channel (Zhao et al., 2001; Lu et al., 2019) and blocks the Ca^2+^-mediated opening of the mitochondria permeability transition pore (MPTP) channel, thus supporting maintenance of mitochondrial membrane potential (Yao et al., 2010; Lu et al., 2019; Papu John et al., 2019). In addition, H_2_S serves as an antioxidant and increases glutathione levels. Further, H_2_S activates the prominent antioxidant master switch, nuclear factor−erythroid 2−related factor 2 (Nrf2), by sulfhydration of cysteine residues of Kelch-like ECH-associated protein 1 (Keap1) dimer. This breaks Keap1 inhibitory binding to Nrf2 and allows for Nrf2 to translocate to the nucleus (Hourihan et al., 2012; Yang et al., 2012), where it activates expression of antioxidant and cytoprotective genes, such as glutathione peroxidase (Gpx), thioredoxin (Trx), superoxide dismutase (SOD), and heme oxygenase-1 (HO-1). H_2_S mediated sulfhydration of other proteins confers additional cell protective effects (Mustafa et al., 2009; Zhao et al., 2014). For example, H_2_S activates the histone deacetylase sirtuin-1 (SIRT1) through its sulfhydration, leading to deacetylation of histones and condensation of chromatin, affecting an array of transcription and nuclear receptors. SIRT1 activation thus influences abundant cellular functions, including stimulation of mitochondrial metabolism and biogenesis, antioxidant pathways, DNA repair and inflammation (Du et al., 2018). ARE, antioxidant response element; Cyt C, cytochrome c, FOXO3, Forkhead box protein O3; PGC-1α, proliferator-activated receptor gamma coactivator 1-alpha. Created with BioRender.com.

Involvement of H_2_S in hibernation, be it as a metabolic suppressor or cell protective agent, requires it to be generated by the animal. In mammals, endogenous H_2_S is produced by three enzymes embedded in the trans-sulfuration pathway, i.e., cystathionine β-synthase (CBS), cystathionine γ-lyase (CSE), and 3-mercaptopyruvate sulfur transferase (3MST). The trans-sulfuration pathway generates H_2_S as a byproduct of methionine to cysteine conversion (reviewed in Zuhra et al., 2020), the latter amino acid being a crucial component of the antioxidant, glutathione. CBS and 3-MST are the major H_2_S producing enzymes in the nervous system (Abe and Kimura, 1996; Bruintjes et al., 2014), whereas CSE is more abundant in the cardiovascular system (Yang et al., 2008). CBS and CSE are also secreted by endothelial cells and hepatocytes, and confers H_2_S production in plasma (Bearden et al., 2010). Next to enzymatic synthesis, endogenous H_2_S may also be derived from “sulfide” pools. Pathways are detailed in Figure 7 (left panels).

Hydrogen sulfide confers a wide array of physiological effects. Firstly, it affects mitochondria in a concentration-dependent manner. In brief, at low concentrations, the prevailing effect is stimulation of oxygen consumption and ATP production, whereas high H_2_S concentrations inhibit mitochondrial respiration. Secondly, H_2_S confers potent antioxidant effects, both directly through scavenging of free radicals and indirectly by upregulation of various antioxidant pathways. Mechanisms of these and additional protective pathways are detailed in Figure 7 (right panels).

#### H_2_S in Hibernation

Despite the booming number of publications on H_2_S over the past decade (Szabo, 2018), there is a surprising paucity of data on the involvement of H_2_S in hibernation. To date only two studies have reported on the subject. Talaei et al. (2012) reported an increase in CBS abundance with increased production of H_2_S in lung tissue of torpid Syrian hamster (*M. auratus*), that was rapidly reversed to summer levels during interbout arousal. Increased H_2_S levels were shown to quench Zn^2+^, and possibly drive torpor related lung remodeling (Talaei et al., 2011b) by lowering activity of Zn^2+^-dependent enzymes including metalloproteases and angiotensin converting enzyme (ACE). In addition, Revsbech et al. (2014) showed a change in H_2_S metabolism in plasma and red blood cells of hibernating Scandinavian brown bears (*Ursus arctos*), with a shift toward higher levels of bound sulfane sulfur (thiosulfate and polysulfides), lower levels of free sulfide (H_2_S and HS^–^) and a likely increase in H_2_S producing capacity in plasma and red blood cells. Furthermore, analysis of plasma metabolomics in hibernating 13-lined squirrels showed substantial regulation of substrates and products of the trans-sulfuration pathway, consistent with increased H_2_S synthesis during late torpor and early arousal (D’Alessandro et al., 2017), as found in hamster lung.

The question is whether there is additional indirect evidence for a role of H_2_S in hibernation. Regarding involvement of H_2_S in suppression of metabolism, i.e., initiation or maintenance of torpor, there is little evidence. It should be noted that the original Blackstone et al. (2005) experiments reporting on H_2_S conferring metabolic suppression in mice were performed under hypoxic conditions (17.5% of O_2_). When the experiment was repeated with 21% O_2_, H_2_S failed to induce hypothermia (Baumgart et al., 2010; Hemelrijk et al., 2018), whereas it was successful at 17.5% O_2_, albeit with a much slower onset (Hemelrijk et al., 2018). Further, exposure to severe hypoxia (5% O_2_) reduced T_b_ close to T_a_ (Hemelrijk et al., 2018). Consequently, the authors attribute the observed hypothermia to hypoxemia induced vasodilation, rather than suppression of metabolism. Such a view may be supported by the absence of H_2_S reduction of metabolism in other, larger animal species (Haouzi et al., 2008; Dirkes et al., 2015). As the above studies employ exogenous administration of H_2_S, differences between species may also arise because of differences in pharmacokinetics and diffusion route length. Thus, a pertinent question is what the effect would be from enhancing endogenous production of H_2_S. Overexpression of CBS and CSE confer longevity in Drosophila (Shaposhnikov et al., 2018), but effects on metabolism were not reported. Further, dopamine treatment strongly increases CBS expression and H_2_S production in cells (Talaei et al., 2011a) and in rats *in vivo* (Dugbartey et al., 2015). However, neither in these experiments, nor in the literature, has a torpor-like reduction of metabolism been noted following treatment with dopamine. Finally, changes in mitochondrial function during torpor have been explored. Compared with arousal, torpor leads to a 40% lower activity of ETC complex I, a 60% lower activity of ETC complex II, but does not affect the activity of ETC complexes III, IV, or V, as measured in isolated mitochondria derived from 13-lined ground squirrel liver (Muleme et al., 2006; Brown et al., 2012; Mathers et al., 2017). Given that metabolic suppression by H_2_S is through inhibition of complex IV, these data do not lend support to a prominent role of H_2_S in induction or maintenance of torpor. However, a finite verdict awaits the development of (inducible) knock-out animals and/or development of selective inhibitors of H_2_S producing enzymes.

On the other hand, cellular protective effects induced by H_2_S may well play a role in hibernation associated antioxidant and cell protective adaptations outlined in previous sections. Notably, consistent with a prominent antioxidant effect of H_2_S (Figure 7), hibernation features a strong upregulation of antioxidant mechanisms through the Nrf2 pathway (Morin et al., 2008; Ni and Storey, 2010; Wu and Storey, 2014; Yin et al., 2016; Frigault et al., 2018; Wei et al., 2018b). These and other studies report upregulation or activation of downstream genes and proteins, including, but not limited to, phosphorylated Nrf2, superoxide dismutase, glutathione reductase and peroxidase, catalase, thioredoxin, and miRNA miR-200a. Further, a number of the above studies document higher levels of glutathione in various organs, which may be compatible with enhanced flux in the trans-sulfuration pathway (D’Alessandro et al., 2017) and a coupled increase in H_2_S production (Figure 7). Interestingly, hibernator cells seem to display cell-autonomous adaptations that enable upkeep of mitochondrial function during cold stress, thus safeguarding ATP production and strongly limiting oxygen radical production, as demonstrated in Syrian hamster kidney cell lines (Hendriks et al., 2017, 2020) and in neurons differentiated from pluripotent stem cells of ground squirrels (Ou et al., 2018), possibly by mild inhibition of complex I. In addition, cooled Syrian hamster kidney cells harnessed their glutathione production, thus effectively precluding ferroptotic cell death (Hendriks et al., 2020). Hibernators have been described to have developed natural resistance to chronic kidney disease (CKD), often associated with cardiovascular impairments in humans (for reviews, see Romagnani et al., 2017; Sarnak et al., 2019), despite their long hibernation fast during winter. Indeed, it was recently reported that markers of CKD differed significantly between animal species with different feeding habits and thermoregulatory behaviors, and that hibernating brown bears and garden dormice displayed higher levels of betaine and choline (known to be cardioprotective) and lower, sometimes even non-detectable, levels of trimethylamine N-oxide (TMAO), a marker of CDK in human and animals (Ebert et al., 2020). Hence, the betaine endogenously produced by the organism may protect organs (notably kidney) of bears, dormice, and other hibernators from oxidative or other damages during the hibernation period of depressed metabolism. Similarly, previous research showed that cooled hibernator kidney cells maintained production of H_2_S and that dopamine induction of CBS precludes cooling-induced cell death in non-hibernator kidney cells (Talaei et al., 2011a). Together, these observations suggest that H_2_S may play a role in the cell-autonomous effects observed in hibernator cells.

Taken together it appears that research efforts into the role of H_2_S in hibernation need to be intensified. To date, the evidence that H_2_S confers entrance or maintenance of torpor is very limited. Conversely, cell protective adaptations by H_2_S have a close resemblance to those documented in hibernation, which makes H_2_S involvement herein likely.

## Conclusion

The flexibility of torpor use as an adaptive strategy enables different heterothermic mammals to substantially suppress their energy requirements during periods of severely reduced food availability. The phenotype of torpor is associated with marked metabolic adaptations, at the whole organism and cellular/molecular levels, that contrast with (i) the behavior of hibernation, e.g., food- vs. fat-storing hibernators, and (ii) the form of hypometabolic responses, i.e., opportunistic (daily) torpor vs. seasonal hibernation, even within one species. The torpid state is also associated with highly efficient rehabilitation and protective mechanisms ensuring continuity of proper functions in the animal, including key organs such as the brain or the heart. There are clear perspectives to be drawn out from the torpor and hibernation phenotypes, notably in terms of models for calorie restriction or intermittent fasting, e.g., fat vs. food storing species, but also for organ (e.g., brain) and tissue resistance to oxidative stress or cell protective adaptations to challenges such as ischemia-reperfusion. Given that most of these mechanisms have been addressed with placental mammals, future work comparing placentals with monotremes, marsupials and birds in a phylogenetic context would reveal convergent evolutionary pathways.

## Author Contributions

All authors drafted and critically revised the manuscript.

## Conflict of Interest

The authors declare that the research was conducted in the absence of any commercial or financial relationships that could be construed as a potential conflict of interest.
